# The Assessment of Prevalence and Predictors of Impostor Phenomenon Feelings Among Dental Students in Croatia and Bosnia and Herzegovina—A Cross‐Sectional Study

**DOI:** 10.1002/hsr2.72873

**Published:** 2026-07-25

**Authors:** Zrinka Biloglav, Ivana Škrlec, Petar Medaković, Ivan Padjen, Tatjana Ružić, Anđelo Kurtin, Vedran Jakupović, Džan Ahmed Jesenković, Simeona Olić, Nicole Stojanović, Eva Mandić, Emanuela Živko, Danijela Marović, Selma Jakupović

**Affiliations:** ^1^ Department of Medical Statistics, Epidemiology and Medical Informatics School of Public Health Andrija Štampar Zagreb Croatia; ^2^ School of Medicine, University of Zagreb Zagreb Croatia; ^3^ Faculty of Dental Medicine and Health Josip Juraj Strossmayer University of Osijek Osijek Croatia; ^4^ Department of Radiology Polyclinic Croatia Zagreb Croatia; ^5^ Division of Clinical Immunology and Rheumatology, Department of Internal Medicine University Hospital Centre Zagreb Zagreb Croatia; ^6^ Department of Psychiatry University Hospital Centre Rijeka Rijeka Croatia; ^7^ Faculty of Health Studies University of Sarajevo Sarajevo Bosnia and Herzegovina; ^8^ Department of Epidemiology and Biostatistics, Faculty of Medicine University of Sarajevo Sarajevo Bosnia and Herzegovina; ^9^ Private Dental Practice Zagreb Croatia; ^10^ School of Dental Medicine University of Zagreb Zagreb Croatia; ^11^ Department of Restorative Dentistry and Endodontics, Faculty of Dentistry University of Sarajevo Sarajevo Bosnia and Herzegovina

**Keywords:** dental students, impostor phenomenon, impostor syndrome, medical students

## Abstract

**Background and Aims:**

The impostor phenomenon (IP) is an individual's internal experience of intellectual phoniness, characterized by an inability to internalize success, leading to self‐doubt and fear of being exposed as a fraud. We examined IP among Croatian and Bosnian‐ Herzegovinian undergraduate dental students.

**Methods:**

A cross‐sectional study (*n* = 679) was conducted at the School of Dental Medicine, University of Zagreb (*n* = 355), and the Faculty of Dentistry, University of Sarajevo (*n* = 324). We aimed to assess the risk associated with certain predictors, such as sociodemographic characteristics, substance use, quality of life, physical and mental health, social interactions, mental health issues, and academic and non‐academic stressors, in relation to a clinically relevant level of impostorism measured by the Clance Impostor Phenomenon Scale (CIPS).

**Results:**

Dental schools in Sarajevo and Zagreb had similar average CIPS scores, 58.5 versus 58.7. Females had significantly higher average scores than males (Sarajevo: 60.1 vs. 53.7; Zagreb: 60.0 vs. 52.4). Every second student had a clinically relevant level of impostor feelings. Transitional years, 1st, 4th, and 6th, exhibited the highest average scores. Alcohol consumption, smoking, parental expectations, PHQ‐9 scores, and lower quality of life increased risk, while social inclusion in the broader community reduced risk.

**Conclusion:**

IP is highly prevalent and influenced by internal and external factors. This study was the first to examine IP among Croatian and Bosnian and Herzegovinian dental students. Dental schools should implement targeted interventions and psychological support into curricula to build student resilience.

## 1 Introduction

The impostor phenomenon (IP), colloquially known as impostor syndrome or impostorism, is a psychological construct first described by psychologists Clance and Imes in the 1970s among high‐achieving women. The Clance Impostor Phenomenon Scale (CIPS) is the most well‐known and widely used assessment tool [1]. Although this scale does not have a standard cut‐off, scores above 60 are considered clinically relevant [2].

Individuals affected by this puzzling phenomenon display a range of associated emotions, attitudes, and behaviors. Impostors often perceive themselves as inadequate, doubt their intelligence and abilities compared to how others see them, and are persistently and irrationally afraid of being exposed as a “fraud”. Despite its prevalence, this distinct form of self‐doubt is not classified as a psychiatric disorder (e.g., DSM‐V criteria). Nevertheless, six core characteristics define this psychological pattern [1, 3, 4]: (i) the impostor cycle, (ii) perfectionism, (iii) the superwoman/superman aspect, (iv) atychiphobia, (v) denial of competence and minimization of praise, and (vi) fear and guilt associated with success [1, 3, 4]. The IP is situated within the mental health continuum and shares clinical similarities with other mental health conditions. Mental health professionals recognize the legitimacy of this phenomenon and its associations with various mental health issues [5]. Its psychological impact includes emotional suffering, psychological distress, persistent dysphoric stress, anxiety, depression, and drug dependence [6, 7]. However, it remains difficult to distinguish causal associations and comorbidity between these mental health issues and impostor feelings.

Impostor feelings are common in the general population and affect up to three‐quarters of people during their lifetime, regardless of gender, age, or background [8, 9]. A systematic review suggests that parental styles and behaviors, attachment styles, maladaptive parenting, parent–child relations, and familial achievement orientation moderately correlate with IP [10]. According to Langford and Clance (1993), IP is associated with personality traits, such as high neuroticism, conscientiousness, and introversion [11]. Clance et al. (1995) noted that interpersonal and social contexts play a role in the formation of IP [12]. Since impostorism negatively impacts the mental and physical well‐being of individuals, particularly high achievers in professions such as healthcare and education, it has become a popular research topic. Its increasing prevalence has encouraged researchers to focus on consolidation, synthesis, and studies of specific populations [13].

Medical students, including those in nursing, pharmacy, and dentistry, are especially prone to impostor feelings. They are more psychologically vulnerable and bear a higher mental burden than their peers. According to Franchi et al., up to 60% are affected by this internal experience [14]. Impostor feelings hinder their personal and career development and their ability to achieve their full potential [15]. Despite growing research, the causes of IP remain unclear. Studies have identified several sociodemographic factors that may affect medical students' psychological adjustment, such as gender, marital status, race, prior mental health history, and academic year [16, 17, 18, 19].

Dental students, in particular, experience increased levels of depression, anxiety, stress, obsessive‐compulsive behaviors, and interpersonal sensitivity [20, 21, 22, 23, 24, 25]. This heightened stress is partly due to the extensive time they spend with patients during their academic training. Stress varies by academic year, but the transition from preclinical to clinical stages can especially trigger feelings of distress and impostorism [26].

Although impostor thoughts have been extensively studied among medical students, few studies have specifically addressed impostorism among dental students [27, 28, 29]. Our study aimed to bridge this gap by focusing exclusively on dental students at public universities in Bosnia and Herzegovina (BiH) and Croatia. The primary objective of this cross‐sectional study was to estimate the prevalence and compare the burden of IP among students at these two universities, which has not been previously evaluated. We hypothesized that the IP prevalence would differ according to gender and academic year. In addition, given the complex and multifaceted nature of IP, we performed an exploratory analysis to identify independent predictors of clinically relevant impostorism, including sociodemographic characteristics, substance use, quality of life, physical and mental health, social interactions, mental health issues, and academic and non‐academic stressors.

## 2 Material and Methods

### 2.1 Study Design and Sampling

A cross‐sectional study was conducted among undergraduate dental students at the School of Dental Medicine, University of Zagreb (2022–2023), where 459 students were invited to participate, and at the Faculty of Dentistry, University of Sarajevo (2023–2024), where 522 students were invited. A convenience sampling method was used, and students were invited to participate in the research via face‐to‐face recruitment during regular classes. They completed an anonymous paper questionnaire designed for the study, which included sociodemographic items, such as parents' education level, relationship status, and self‐assessed financial status, accommodation, and working hours per week. In Bosnia and Herzegovina, the questionnaire was tailored to local characteristics and adapted to the Bosnian/Croatian/Serbian language.

Self‐assessment of quality of life, physical and mental health, and social interaction was measured on a Likert scale ranging from 1 (far below average) to 5 (far above average). To assess anxiety and depression, we used General Anxiety Disorder‐7 (GAD‐7) and Patient Health Questionnaire‐9 (PHQ‐9), previously validated in the Croatian [30] and Bosnian populations [31]. GAD‐7 evaluates the symptoms of anxiety occurring during the 2 weeks before completion and is used as a screening for generalized anxiety disorder. The PHQ‐9 is a validated instrument for depression screening. It consists of nine questions with responses on a four‐point scale, each providing information about one criterion for depression defined by DSM‐IV [32]. In this analysis, for PHQ‐9 and GAD‐7, we did not use a cut‐off value of ≥ 10, which indicates clinically significant depression and anxiety, but instead used the total score.

Academic and non‐academic stressors were rated from 1 to 5, with 1 meaning “not stressful at all” and 5 meaning “extremely stressful.”

To measure IP, we used the 20‐item CIPS questionnaire [1, 33, 34]. Each question offered multiple‐choice options: “not at all true,” “rarely,” “sometimes,” “often,” and “very true.” Each response was assigned to a numerical value from 1 to 5, respectively. These values were summed to produce total scores ranging from 20 to 100, which were used to classify each participant into one of the four levels representing the intensity of impostor feelings: few (40 or less), moderate (41 to 60), frequent (61 to 80), or intense (81 or more) [35]. Scores of 61 or higher indicate high levels of impostorism, while scores of 60 or below indicate non‐impostors [2].

### 2.2 Ethical Considerations

This study fully conformed to the principles of the Declaration of Helsinki. After the study aims and content were presented, all participants provided written consent before participating. Ethical approval was obtained from the School of Dental Medicine, University of Zagreb (05‐PA‐30‐22‐11/2023), and the Faculty of Dentistry, University of Sarajevo (02‐3‐4‐19‐3‐2/2024).

### 2.3 Statistical Analyses

We used descriptive statistics to estimate means (M), standard deviations (SD), medians (Mdn), and interquartile ranges (IQR) for continuous variables and counts and percentages for categorical variables. Based on the normality of distribution assessed with the Shapiro–Wilk test, we applied appropriate statistical tests. A chi‐square test of independence was conducted to examine the relationship between categorical variables. One‐way ANOVA or the Kruskal–Wallis test was used to assess differences in average CIPS scores across academic years with effect size (η^2^). To compare median CIPS values between genders, we used the Mann–Whitney test with effect size (r). We used a Spearman rank‐order correlation to examine the association between CIPS score and age in the total sample. All tests were two‐sided.

We used the enter‐method logistic regression model to assess the association between student characteristics included as covariates and clinically significant CIPS. Results are reported as odds ratios (OR) with 95% confidence intervals (CI). We calculated the Nagelkerke R^2^ [36]. The variance inflation factor (VIF) was estimated to detect multicollinearity in the regression analysis. A VIF value of 5 to 10 indicates potential multicollinearity [37], suggesting dependence among multiple independent variables in the model and possible effects on the interpretation of the regression results.

All comparisons were two‐tailed, with *p* < 0.05 considered significant. All analyses were conducted in JASP version 0.97.1 [38].

## 3 Results

Among 679 students, 535 were female, and 144 were male. The response rates in Sarajevo and Zagreb were 70.59% (*n* = 324) and 68.00% (*n* = 355), respectively. Females outnumbered males in Sarajevo, 75.31% versus 24.69%, and Zagreb, 81.97% versus 18.03%. Sociodemographic characteristics by university are presented in Table 1.

**Table 1:** Characteristics of dental students according to universities.

Variable	Sarajevo	Zagreb	*p*
Socio‐demographic characteristics			
Male	*n* = 80	*n* = 64	χ^2^ (1, 144) = 1.79 *p* = 0.18
Female	*n* = 244	*n* = 291	χ^2^ (1, 535) = 4.13 *p* = 0.04
Age range (years)	19–29	19–28	
Total (years)	M = 22.18 SD = 2.28 Mdn = 22.00, IQR (22.00 to 24.00)	M = 21.58 SD = 1.95 Mdn = 22.00, IQR (20.00 to 23.00)	*U* = 64889 *p* = 0.004 *r* = −0.13
Males (years)	M = 22.66, SD = 2.43 Mdn = 22.00 IQR (21.00–25.00)	M = 21.36, SD = 1.66 Mdn = 21.00 IQR (20.00–23.00)	*U* = 3285 *p* = 0.003 *r* = −0.28
Females, mean age, (SD)	M = 22.02, SD = 2.22 Mdn = 22.00 IQR (20.00–23.00)	M = 21.63, SD = 2.02 Mdn = 22.00 IQR (20.00–23.00)	*U* = 38351.50 *p* = 0.11 *r* = −0.08
Relationship status			
Married	10.19%, *n* = 33	4.51%, *n* = 16	χ^2^ (1, 49) = 5.89 *p* = 0.02
Partnership	20.06%, *n* = 65	34.37%, *n* = 122	χ^2^ (1, 187) = 17.37 *p* < 0.001
Single	60.75%, *n* = 226	61.13%, *n* = 217	χ^2^ (1, 443) = 0.18 *p* = 0.67
Financial status			
Above average	43.52%, *n* = 141	49.86%, *n* = 177	χ^2^ (1, 318) = 4.08 *p* = 0.04
Average	50.31%, *n* = 163	46.48%, *n* = 165	χ^2^ (1, 328) = 0.01 *p* = 0.91
Below average	6.17%, *n* = 20	3.66%, *n* = 13	χ^2^ (1, 33) = 1.48 *p* = 0.22
Alcohol and smoking			
Alcohol consumption (*n*, %)			
Never	*n* = 219, 67.59%	*n* = 20, 5.63%	χ^2^ (1, 239) = 165.69 *p* < 0.001
Rarely or sometimes	*n* = 87, 26.85%	*n* = 323, 90.99%	χ^2^ (1, 410) = 135.84 *p* < 0.001
Often or very often	*n* = 18, 4.94%	*n* = 12, 3.38%	χ^2^ (1, 30) = 0.57 *p* = 0.45
Daily	*n* = 2, 0.62%	*n* = 0, 0%	na
Smoking			
Current smoker	*n* = 91, 28.09%	*n* = 59, 16.62%	χ^2^ (1, 150) = 6.83 *p* = 0.009
Former smoker	*n* = 16, 4.94%	*n* = 18, 5.07%	χ^2^ (1, 34) = 0.12 *p* = 0.73
Non‐smoker	*n* = 217, 66.98%	*n* = 278, 78.31%	χ^2^ (1, 495) = 7.52 *p* = 0.006
Self‐assessment of quality of life, physical and mental health, and social interactions			
Quality of life	M = 3.81, SD = 0.83 Mdn = 4.00, IQR (3, 4)	M = 4.04, SD = 0.71 Mdn = 4.00, IQR (4, 5)	*U* = 48959 *r* = 0.15 *p* < 0.001
Mental health	M = 3.79, SD = 0.94 Mdn = 4.00, IQR (3, 4)	M = 3.85, SD = 0.83 Mdn = 4.00, IQR (3, 4)	*U* = 56212 *r* = 0.02 *p* = 0.59
Physical health	M = 3.97, SD = 0.88 Mdn = 4.00, IQR (3, 5)	M = 3.98, SD = 0.75 Mdn = 4.00, IQR (3, 5)	*U* = 58603 *r* = −0.02 *p* = 0.65
Social inclusion in broader community	M = 3.51, SD = 1.06 Mdn = 4.00, IQR (3, 4)	M = 3.81, SD = 0.90 Mdn = 4.00, IQR (3, 4)	*U* = 48745.50 *r* = 0.15 *p* < 0.001
Interpersonal relationship with close social environment	M = 4.19, SD = 0.92 Mdn = 4.00, IQR (4, 5)	M = 4.29, SD = 0.73 Mdn = 4.00, IQR (4, 5)	*U* = 56127.50 *r* = 0.02 *p* = 0.56
Anxiety and depression			
GAD‐7	Range (0–21) M = 7.94, SD = 5.30 Mdn = 7.00, IQR (4.00, 12.00)	Range (0–20) M = 6.03, SD = 4.29 Mdn = 5.00, IQR (3.00, 8.00)	*U* = 69384 *r* = −0.21 *p* < 0.001
PHQ‐9	Range (0–27) M = 11.17, SD = 6.66 Mdn = 10.00, IQR (6.00, 16.00)	Range (0–26) M = 7.84, SD = 4.83 Mdn = 7.00, IQR (4.00, 10.50)	*U* = 74489 *r* = −0.29 *p* < 0.001
Academic and non‐academic stressors			
Structure and organization of the study program	M = 3.13, SD = 1.03 Mdn = 3.00, IQR (2, 4)	M = 2.83, SD = 0.85 Mdn = 3.00, IQR (2, 3)	*U* = 68037.50 *r* = −0.18 *p* < 0.001
Career concerns	M = 3.49, SD = 1.30 Mdn = 4, IQR (3, 5)	M = 3.13, SD = 1.05 Mdn = 3, IQR (2, 4)	*U* = 68306.50 *r* = −0.19 *p* < 0.001
Concern for future quality of life	M = 3.48, SD = 1.25 Mdn = 4, IQR (3, 5)	M = 3.16, SD = 1.11 Mdn = 3, IQR (2, 4)	*U* = 66809.50 *r* = −0.16 *p* < 0.001
Lack of free time	M = 3.20, SD = 1.26 Mdn = 3, IQR (2, 4)	M = 2.99, SD = 1.14 Mdn = 3, IQR (2, 4)	*U* = 63214.50 *r* = −0.09 *p* = 0.02
Parental expectation	M = 2.47, SD = 1.38 Mdn = 2, IQR (1, 3)	M = 1.98, SD = 1.14 Mdn = 2, IQR (1, 3)	*U* = 68435 *r* = −0.19 *p* < 0.001

Abbreviations: GAD‐7, General Anxiety Disorder‐7; IQR, interquartile range; M, mean; Mdn, median; n, number; na, not assigned; p, level of statistical significance; PHQ‐9, Patient Health Questionnaire‐9; r, effect size; U, test statistics.

A Mann–Whitney *U* test revealed no statistically significant difference in CIPS scores between Sarajevo (*M* = 58.48, *n* = 324) and Zagreb (*M* = 58.66, *n* = 355, *U* = 57497, *p* > 0.99, *r* = 0.0002).

**Figure 1:**
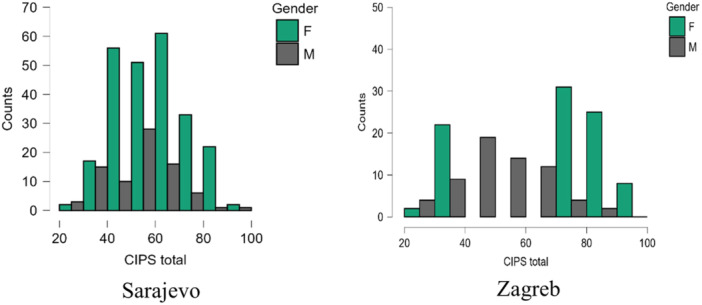
Distribution of CIPS scores according to university and gender. CIPS = Clance Impostor Phenomenon, F = females, M = males, Scale,. Sarajevo, Zagreb.

CIPS scores by university and gender are shown in Figure 1. In Sarajevo, females (*M* = 60.06, *n* = 244) had significantly higher average CIPS scores than males (*M* = 53.66, *n* = 80, *U* = 7458, *p* = 0.002, *r* = 0.24). In Zagreb, females (*M* = 60.03, *n* = 291) also had significantly higher average CIPS scores than males (*M* = 52.4, *n* = 64, *U* = 6707, *p* < 0.001, *r* = 0.28).

Students were categorized by CIPS level, gender, and academic year (Table 2). Few impostor feelings were more prevalent among males than females, with prevalence ratios of 2.4 in Zagreb and 2.8 in Sarajevo. In contrast, intense impostor feelings were more prevalent among females than males, 3.6 times in Zagreb and 3.9 times in Sarajevo. In the total sample, non‐impostors significantly outnumbered impostors, 56% (*n* = 382) versus 44% (*n* = 297), χ^2^ = 10.64 (df = 1, *N* = 679), *r* = 0.64, *p* = 0.001.

**Table 2:** CIPS levels according to gender and university.

	CIPS level	University of Zagreb			University of Sarajevo		
		Male	Female	Total	Male	Female	Total
Non‐impostors	Few	13 20.31%	24 8.25%	37 10.42%	18 22.50%	19 7.79%	37 11.42%
	Moderate	16 25.00%	104 35.74%	120 33.80%	22 27.50%	94 38.52%	116 35.80%
Impostors	Frequent	33 51.56%	130 44.67%	163 45.92%	38 47.50%	107 43.85%	145 44.75%
	Intense	2 3.13%	33 11.34%	35 9.86%	2 2.50%	24 9.84%	26 8.02%
	Total	64 100%	291 100%	355 100%	80 100%	244 100%	324 100%

There were significantly more non‐impostors than impostors in Sarajevo, 56.17% (*n* = 182) versus 43.83% (*n* = 142), χ^2^ = 4.94 (df = 1, *N* = 324, *p* = 0.03), and in Zagreb, 56.34% (*n* = 200) versus 43.66% (*n* = 155), χ^2^ = 5.70 (df = 1, *N* = 355, *p* = 0.02).

Sarajevo and Zagreb had similar proportions of non‐impostors and impostors χ^2^ = 0.002, (df = 1, *N* = 679, *p* = 0.97). Moreover, we compared the prevalence of impostors and non‐impostors by sex.

Among all males (*n* = 144), there were significantly more non‐impostors, 70.83% (*n* = 102), than impostors, 29.17% (*n* = 42) χ^2^ = 25, (df = 1, *N* = 144, *p* < 0.001). In Sarajevo, the proportions were 70% (*n* = 56) non‐impostors versus 30% (*n* = 24) impostors χ^2^ = 12.80, (df = 1, *N* = 80, *p* < 0.001), and in Zagreb, 71.88% (*n* = 46) non‐impostors versus 28.13% (*n* = 18) impostors χ^2^ = 12.25 (df = 1, *N* = 64), *p* < 0.001.

Among all females (*n* = 535), 52.34% (*n* = 280) were non‐impostors and 47.66% (*n* = 255) were impostors χ^2^ = 1.17, (df = 1, *N* = 525), *p* = 0.28. In Sarajevo, 51.64% (*n* = 126) were non‐impostors and 48.36% (*n* = 118) were impostors χ^2^ = 0.26, (df = 1, *N* = 244), *p* = 0.61. In Zagreb, 52.92% (*n* = 154) were non‐impostors and 47.08% (*n* = 137) were impostors χ^2^ = 0.99, (df = 1, *N* = 291), *p* = 0.32.

**Figure 2:**
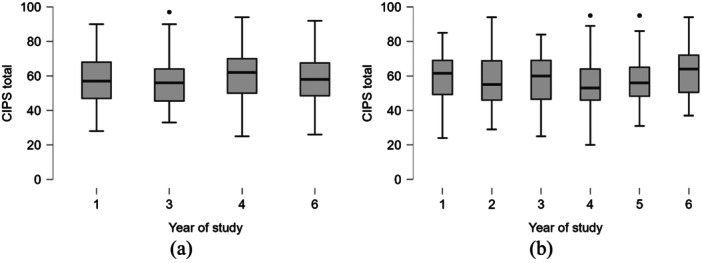
Average CIPS value across academic years – total: (a) Sarajevo and (b) Zagreb.

A one‐way ANOVA was conducted to determine if there are differences among average CIPS values for Sarajevo and Zagreb across academic years (Figure 2a, Figure 2b). Average CIPS scores across academic years were similar in Zagreb *F* (5, 349) = 1.93, *p* = 0.09, η^2^ = 0.03 and in Sarajevo *F* (3, 320) = 2.67, *p* = 0.05, η^2^ = 0.02.

In Sarajevo, both males and females had the highest average CIPS score in the 4th year, followed by the 1st year, but there were no significant differences across academic years for males *F* (3, 76) = 1.69, *p* = 0.61, η^2^ = 0.02 or females *F* (3, 240) = 1.99, *p* = 0.12, η^2^ = 0.02. However, in the 6th year, females had a significantly higher average CIPS score than males (Table 3).

**Table 3:** Average cips levels across academic years according to gender and university.

Year	Sarajevo			Zagreb		
	Male	Females	*p*	Male	Females	*p*
1st	*n* = 19 Range (28, 76) M = 53.32 SD = 11.91	*n* = 70 Range (29, 90) M = 59.94, SD = 15.09	*t*(87) = −1.79 *d* = −0.46 *p* = 0.08	*n* = 10 Range (24, 70) M = 55.50 SD = 15.59	*n* = 56 Range (33, 85) M = 60.62 SD = 13.23	*t*(64) = −1.09 *d* = −0.38 *p* = 0.28
2nd				*n* = 9 Range (36, 81) M = 57.00 SD = 17.71	*n* = 51 Range (29, 94) M = 58.61 SD = 16.62	*t*(58) = −0.27 *d* = −0.097 *p* = 0.74
3rd	*n* = 23 Range (34, 88) M = 52.69 SD = 13.09	*n* = 64 Range (33, 97) M = 56.89, SD = 14.11	*t*(85) = −1.25 *d* = −0.30 *p* = 0.22	*n* = 18 Range (25, 70) M = 48.94 SD = 13.37	*n* = 38 Range (33, 84) M = 61.60, SD = 14.16	*t*(54) = −3.18 *d* = −0.91 *p* = 0.002
4th	*n* = 14 Range (25, 94) M = 58.21 SD = 19.14	*n* = 59 Range (36, 89) M = 63.14 SD = 13.45	*t*(71) = −1.13 *d* = −0.34 *p* = 0.26	*n* = 11 Range (20, 89) M = 52.27 SD = 16.96	*n* = 49 Range (26, 95) M = 59.28, SD = 14.60	*t*(58) = −0.78 *d* = −0.26 *p* = 0.44
5th				*n* = 11 Range (39, 74) M = 49.27 SD = 10.03	*n* = 47 Range (21, 95) M = 59.277 SD = 14.60	*t*(56) = −2.15 *d* = −0.72 *p* = 0.04
6th	*n* = 24 Range (26, 70) M = 52.21 SD = 13.52	*n* = 51 Range (34, 92) M = 60.65 SD = 14.19	*t*(73) = −2.44 *d* = −0.60 *p* = 0.02	*n* = 5 Range (44, 76) M = 57.60 SD = 11.80	*n* = 50 Range (37, 94) M = 4.08 SD = 15.21	*t*(56) = −2.15 *d* = −0.72 *p* = 0.36
	*F* (3, 76) = 0.60 η^2^ = 0.02 *p* = 0.61	*F* (3, 240) = 1.99 η^2^ = 0.02 *p* = 0.12		*F* (5, 58) = 0.71 η^2^ = 0.06 *p* = 0.62	*F* (5, 285) = 1.63 η^2^ = 0.03 *p* = 0.15	

Abbreviations: d, Cohen d; M, mean; P, level of statistical significance; SD, standard deviation; η^2^, effect size.

In Zagreb, males had the highest average CIPS score in the 6th year, followed by the 2nd year, but differences across academic years were not significant *F* (5, 58) = 0.71, *p* = 0.62, η^2^ = 0.06. Females had the highest score in the 6th year, followed by the 3rd year, but the differences across years were not significant *F* (5, 285) = 1.63, *p* = 0.15, η^2^ = 0.03. In the 3rd and 5th years, females had significantly higher average CIPS scores than males (Table 3).

In the total sample (*n* = 679), CIPS scores increased with age, rho = 0.02, but this association was not significant (*p* = 0.61).

According to the logistic regression model (Table 4), rare or occasional alcohol consumption, quality of life, PHQ‐9, and parental expectations are significant risk factors for the impostor phenomenon (CIPS > 60), while social inclusion in the broader community and smoking have a protective effect. This model demonstrated moderate explanatory power (Nagelkerke's R^2^ = 0.35), and all predictors showed acceptable levels of multicollinearity. The variance inflation factor (VIF) ranged from 1.05 to 2.26.

**Table 4:** Logistic regression with cips > 60 as a dependent variable.

	Estimate	SE	OR 95% CI	*p*
(Intercept)	−3.37	1.01	0.03 (0.00−0.25)	< 0.001
Sex (males)	0.35	0.25	1.42 (0.88−2.30)	0.15
Relationship status				
Partnership or married			1	
Single	0.02	0.19	1.02 (0.67−1.49)	0.91
Financial status				
Below average			1	
Average	−0.28	0.45	0.76 (0.31−1.84)	0.54
Above average	−0.35	0.45	0.71 (0.29−1.71)	0.44
Alcohol consumption				
Never			1	
Rarely or sometimes	0.51	0.21	1.67 (1.10−2.53)	0.02
Often or very often or daily	−0.35	0.50	0.71 (0.26−1.90)	0.49
Smoking				
Non‐smoker			1	
Former smoker	−0.29	0.43	0.75 (0.32−1.73)	0.49
Smoker	−0.79	0.25	0.46 (0.28−0.74)	0.001
Quality of life	0.36	0.16	1.44 (1.05−1.96)	0.02
Mental health	−0.09	0.14	0.92 (0.69−1.21)	0.54
Physical health	0.01	0.14	1.01 (0.77−1.33)	0.95
Social inclusion in the broader community	−0.39	0.12	0.68 (0.54−0.85)	0.001
Interpersonal relationship with close social environment	0.14	0.14	1.15 (0.88−1.52)	0.31
GAD‐7	0.06	0.03	1.06 (1.00−1.12)	0.06
PHQ‐9	0.15	0.03	1.16 (1.10−1.22)	0.001
Structure and organization of the study program	0.05	0.11	1.05 (0.85−1.31)	0.64
Career concerns	0.04	0.14	1.04 (0.80−1.36)	0.76
Concern for future quality of life	0.21	0.14	1.23 (0.93−1.62)	0.15
Lack of free time	−0.15	0.10	0.86 (0.72−1.04)	0.13
Parental expectation	0.17	0.08	1.18 (1.01−1.38)	0.04

Abbreviations: CI, confidence interval; OR, odds ratio; SE, standard error.

## 4 Discussion

This study of Croatian and Bosnian dental students was the first to analyze the burden of the impostor phenomenon and its association with sociodemographic factors, depression, and anxiety. Results indicate that significantly more students are non‐impostors; nevertheless, 44% of the students can be defined as impostors. We confirmed gender differences, with females experiencing more impostor feelings.

The feminization of medicine, defined as the increased presence of women in traditionally male‐dominated professions, was observed at both dental schools [39, 40]. Societal and policy changes, along with shifts in traditional gender roles, have contributed to this trend. Women outnumbered men, comprising 75.31% of students in Sarajevo and 81.97% in Zagreb, which is higher than in the United States, where women make up half of all dental students [40]. Although medicine was historically considered a male‐dominated field, this perception has changed in many countries, including Croatia [41] and Bosnia [42]. The European Commission's 2020 report addressed the predominance of women among healthcare workers in Bosnia and Herzegovina and Croatia [43]. The proportion of women in dentistry has also increased steadily over the past four decades [44, 45].

Students in Sarajevo and Zagreb differed in the analyzed characteristics. There was a higher proportion of females in Zagreb, while students in Sarajevo were older, with men accounting for this difference (Table 1). However, all students belong to Generation Z and most likely share the same values [46]. More students in Sarajevo were married, but at both universities, up to two‐thirds were single. Previous research also indicated that Gen Z is more likely to delay marriage than previous generations [47]. There were also differences in socioeconomic status. In Zagreb, a higher proportion of students reported above‐average financial situations (Table 1). This may be due to differences in socioeconomic development, as BiH is classified as a middle‐income country, while Croatia is a high‐income country.

Alcohol consumption varied significantly between dental schools: 90.99% of Zagreb students occasionally consume alcohol, while 67.59% of Sarajevo students never drink alcohol (Table 1). This contrasts with earlier Bosnian studies, which reported abstinence rates of 52.0% and 37.7% [48, 49].

Smoking prevalence is higher among students in Sarajevo than in Zagreb, at 28.09% versus 16.62%. Similar rates have been reported among Bosnian medical students in Banja Luka (27.8%) [50], Croatian medical students (> 30%) [51, 52], and across European countries (30%) [53], with one in five medical students globally being a smoker [54]. Both rates remain below those in their respective general populations, 28.09% versus 35.1% in BiH [55] and 16.62% versus 32.6% in Croatia [56]. The elevated smoking rate among Bosnian students may reflect a coping mechanism, given that two‐thirds of students never drink alcohol [57] (Table 1).

Students in Zagreb have a significantly better quality of life, physical and mental health, and social interactions than students in Sarajevo (Table 1), consistent with significantly higher average scores for anxiety (GAD‐7) and depression (PHQ‐9). This aligns with previous findings of a 30.1% prevalence of depressive symptoms among Sarajevo University students [58].

We also observed significant differences between academic and non‐academic stressors. Among Gen Z dental students, the highest stressors in both Sarajevo and Zagreb were career concerns (3.49 vs. 3.13), future quality of life (3.48 vs. 3.16), and lack of free time (3.20 vs. 2.99). Although academic stressors, including study program structure, were higher in Sarajevo, they remained lower than non‐academic stressors in both dental schools (3.13 vs. 2.83). This reflects Gen Z's typically high career expectations, emphasis on work‐life balance, and greater focus on mental health compared to previous generations [59] (Table 1).

The IP affected students in Zagreb and Sarajevo equally, with mean CIPS scores of 58.66 and 58.48, respectively. Mean CIPS scores ranged from 45.0 to 65.2 across different levels of medical training in various countries [27, 29, 60, 61, 62, 63]. Compared with this range, the mean CIPS values in this study fall into the upper tercile. However, these CIPS values are lower than the average CIPS score of 65 reported among 753 Harvard medical and dental students [64].

In this study, impostorism appears more prevalent among females. Females had higher CIPS scores than males in Sarajevo (60.06 vs. 53.66) and Zagreb (60.03 vs. 52.41), consistent with earlier research identifying female gender as a significant predictor of intense IP [65]. Holliday et al. (2020) reported that medical and dental students were twice as likely to experience intense impostor feelings [64], while Bravata et al. (2020) found that about 50% of studies worldwide reported significantly higher impostor feelings among females [66]. However, a study of medical students in Turkey found no significant gender differences in the frequency of the impostor phenomenon [67]. Stratified analysis by CIPS categories showed that males had a 2.4‐ and 2.8‐times higher proportion of few impostor feelings, while intense impostor feelings were 3.6 and 3.9 times more prevalent among females.

Across the total sample, non‐impostors significantly outnumbered impostors, 56% versus 44%; in Sarajevo, 56.17% versus 43.83%; and in Zagreb, 56.34% versus 43.66%. Notably, approximately every second student met the threshold for clinically relevant impostor phenomenon [68] (Table 2), consistent with Daniels' (2021) finding that nearly 60% of dental students experience clinically relevant impostor feelings affecting daily life [69]. Females were more affected by intense impostor feelings in both populations (Table 2). The findings align with Clance and Imes' (1978) foundational assertion about females' predisposition to impostor experience [3], with gender recognized as a key etiological factor [3, 70]. Although the rates of intense impostor feelings in Zagreb (3.13% males, 11.34% females) and Sarajevo (2.50% males, 9.84% females) are below those reported by Holliday et al. (2020) among Harvard medical and dental students (11% males, 18% females) [64], this finding is corroborated by Henning et al. [29]. However, interpretation requires caution given small male sample sizes in some CIPS categories [28]. When discussing gender and IP, some other issues should also be discussed.

First, it should be noted that the impact of the female gender is not exclusively associated with IP. Research suggests that anxiety, depression, and low self‐esteem are more prevalent in females [7, 30, 71]. Females have a 70% higher lifetime prevalence of major depressive episodes than men [72]. In addition to biological differences, females' higher risk of developing mood disorders and anxiety is attributable to their subjective perception of experiencing greater stress and less favorable cognitive assessments, such as trouble remembering and learning new things and difficulty making everyday decisions [73, 74, 75]. Social status, roles, and access to resources, options, and treatment can also help explain the prevalence of mental health issues among genders.

Perfectionism is more common among females and is a key driver of IP [76]. Henning et al. confirmed associations between perfectionism, distress, and impostor feelings among health profession students (*n* = 477) [29]. Females face higher social expectations and pressure and are more susceptible to negative attribution styles, often undervaluing their abilities and attributing success to luck rather than competence [76, 77, 78]. Gender also shapes impostor response patterns: males react more negatively to accountability [79], while females focus on normative comparison, in contrast to males' failure‐avoidance orientation [80]. Whether IP is a fixed personality trait or a malleable state amenable to mentoring or counseling remains debated [81], with cultural context also implicated [82].

The social perspective on gender inequality deserves consideration alongside the psychological perspective. Croatia scored below the EU average on the Gender Equality Index (GEI), with particularly pronounced inequalities in the knowledge domain [83], although a GEI score for BiH was unavailable [84].

Institutional factors within medical culture increase the prevalence of impostorism. Mistreatment is common, affecting 4% to 41% of students, with up to 83% of dental students reporting at least one form of mistreatment, mostly psychological, by classmates, and about one‐third experiencing sexual harassment [85]. This toxic medical culture exacerbates impostor feelings and perfectionism, particularly among high‐achievers [86, 87]. In contrast, mentorship can mitigate impostor feelings and foster positive self‐perceptions [88], but its availability and effectiveness for female students remain limited, and they report lower clinical self‐confidence [89]. Dental education is inherently demanding, and although impostor phenomenon may fluctuate throughout the curriculum [65], most studies report an inconsistent association with academic year [61]. However, increased psychological distress and a decline in mental health have been documented during the transition from preclinical to clinical training and in the final year before starting residency [87, 90].

Students in Zagreb and Sarajevo had similar average CIPS scores, but trends varied by academic year and gender (Figures 1 and 2). The transitional years of the curriculum at both universities appear to be more affected by the impostor phenomenon. In Zagreb, both genders reached the highest average CIPS values in the final 6th year. After that year, impostor traits mostly affected females in the 1st and 4th years, while males had high levels in the 2nd and 1st years (Table 3). In Sarajevo, females and males showed a more uniform trend; CIPS scores were highest in the 4th year and, after that, in the 1st year (Table 3). According to this study, the average CIPS scores among 1st year dental students in Zagreb are the highest among females and the second highest among males and females in Sarajevo and Zagreb, respectively. This is not unexpected, as new students are highly stressed, which can negatively affect academic performance and mental and physical health [91]. Stressful transitions from high school can trigger impostor thoughts [92]. Additionally, first‐year stressors in both curricula, such as anatomy dissection exercises, high workloads, frequent evaluations, and exposure to equally high‐achieving peers, create fertile ground for impostor experiences [93]. Additional stressors include financial problems, language and cultural barriers for international students, and clinical‐year concerns about work quality and misalignment between self‐expectations and external perceptions [94]. Furthermore, the 1st year students are uprooted in family and friends and often worry about academic performance, particularly if they pay tuition. This challenge especially affects students with academic struggles. As mentioned earlier, IP among 1st year students is likely a consequence of facing their future careers. For dental students, the specific association between psychological adjustment, age, and academic year suggests higher distress among students in the earlier academic years [29]. Traditionally, during the 4th year, students are introduced to clinical responsibilities and firsthand experience of medical stressors. Students at the beginning of clinical clerkships in Sarajevo have the highest average CIPS scores (Figure 2a). The preclinical to clinical transition is considered the most critical phase of dental and medical education [28, 95, 96, 97, 98]. Unlike Zagreb, where students maintain stable peer groups, Sarajevo students form new groups for clinical rotations, losing peer support networks. Clinical training intensifies impostor feelings by exposing knowledge deficits, demanding real patient care, and incorporating pimping culture, particularly its malignant form, which can severely undermine self‐esteem and reinforce feelings of fraudulence. There is also significant stress throughout the entire 4‐year academic and clinical program in dental schools [99, 100]. Murphy et al. found greater perceived stress in dental students due to feelings of professional inadequacy and impostorism, as dentistry demands excellent precision and strict technical requirements [26]. An unstructured learning environment, lack of free time, financial concerns, demanding assignments, student abuse, and exposure to human suffering can be additional sources of distress [93]. Transitioning to a new role can trigger impostor feelings in anyone. In Zagreb, the 6th year represents the peak of impostorism for both genders (Figure 2b). Final‐year students face compounded stressors, such as high‐stakes examinations, career uncertainty, and anticipation of independent clinical decision‐making, contributing to elevated impostor feelings. Notably, impostor feelings appear to diminish after graduation, as clinical experience and internships foster workplace confidence [101]. Evidence on trends during the curriculum remains mixed, however, with Sawant et al. reporting significantly higher CIPS scores in 1st‐year versus final‐year students [102].

Rare or occasional alcohol consumption significantly increased the risk of IP by 67%, which is not surprising since it may serve as a coping mechanism for negative feelings [103, 104]. Better quality of life also represents a risk factor (OR = 1.44). Parental expectations increase risk by 18%, most likely through a complex relationship with impostorism. High parental expectations become harmful when associated with criticism or conditional regard. In a medical education cohort, 72% of those with impostor syndrome self‐identified parental expectations as a contributor, especially among perfectionists [105, 106, 107]. The PHQ‐9 score, a proxy for depression, is a slightly lower risk factor than parental expectations (OR = 1.16). A growing body of research shows that impostor feelings deteriorate mental health, predominantly expressed as increased anxiety and depression, low self‐esteem, and an external locus of control [66, 108, 109, 110, 111, 112, 113, 114]. Anxiety is closely related to IP [66]. Smoking and social inclusion in the broader community were protective factors, decreasing the risk of impostorism by 54% and 32%, respectively. Since participants' social anxiety was positively correlated with their impostor expressions, it is not surprising that social inclusion in the broader community played a significant protective role (Table 4). Gender did not significantly affect IP, although the most recent meta‐analysis showed that women experience more impostor feelings, but the effect is moderate [115]. Socio‐economic status was also found to be unrelated to IP, which aligns with a previous study that reported this finding as nuanced and sometimes mixed [116]. Moreover, some research suggests that some of the previously mentioned factors may also act as mediators in certain associations between IP and other related variables.

Zagreb and Sarajevo dental students exhibit a similar prevalence of IP, but there is a significant association between students' gender and impostor feelings. Females are more likely to experience this psychological pattern. The potential influence of hidden confounders cannot be excluded with a cross‐sectional study design. Findings from this study support the idea that multiple factors contribute to impostor feelings, including family, peer, and school relationships; institutional and social environments; and expectations. Therefore, extensive qualitative and quantitative research is needed to determine the true burden and causal factors of IP among dental students. Recognizing and addressing both personal and environmental aspects is essential for developing effective strategies to mitigate the effects of IP.

### 4.1 Study Strengths and Limitations

This study provided valuable insights into the prevalence of IP and demonstrated several notable strengths. The major strength is the sample size, which is larger than that of a previous study of dental students in Pakistan (*n* = 203) [101].

The overall response rates in Sarajevo and Zagreb were 70.59% and 68.00%, indicating high survey data accuracy [117]. In this study, a convenience sampling method was used. Although this approach may have introduced selection bias, it is likely to have been non‐differential. There is no evidence to suggest that the variables of interest were systematically distributed differently between students who participated in the study and those who were absent during data collection. Besides a large sample size, this study included the largest dental schools in the selected countries, increasing the representativeness of student experiences at other dental schools. The study also used CIPS, the most widely used instrument for identifying IP [1]. We also acknowledge several limitations that warrant consideration. First, associations should be interpreted cautiously, as a cross‐sectional study design does not allow for the evaluation of causal relationships [118, 119]. Prevalence studies are susceptible to actual or potential bias due to difficulties in obtaining representative samples and inaccuracies in data collection. Additionally, the inability to account for unobservable confounding is a significant limitation. A longitudinal design would likely provide a more in‐depth understanding of this complex behavioral phenomenon, as it accounts for multiple confounding variables. Following students throughout their academic education and adjusting for potential confounders would clarify the true dynamics of impostor feelings. Second, CIPS is a self‐reported questionnaire prone to social desirability bias, in which participants overreport socially desirable behaviors and underreport undesirable ones [120]. Third, despite the high response rate, we cannot estimate how students' willingness to participate influenced the results. Differences in the impostor prevalence of the impostor phenomenon between participating and non‐participating students could not be examined. Fourth, both studies were conducted at a single center. Because institutional and cultural contexts may shape experiences of impostorism, the findings may not be generalized to students at other dental schools. Fifth, we compared impostor prevalence between two universities and across academic years; however, educational programs may differ, so comparisons require caution. Sixth, frequent psychological comorbidities in young people, such as anxiety and depression, were not considered. These comorbidities can increase dropout from the study and decrease the true prevalence of IP. Seventh, having a larger number of dental students at some impostor level would increase the study's accuracy. Some associations may reach significance with an increased sample size. Eighth, there is a lack of normative data for CIPS among the general population and specific student subpopulations, which limits comparisons [2, 121]. Ninth, students in Sarajevo and Zagreb paid no tuition, but data on this were unavailable. Such social disadvantages or privileges could affect the intensity of impostor feelings. Tenth, we did not explore the discriminated social groups, such as ethnic minorities. This is especially relevant for multiethnic Bosnia and Herzegovina [122]. Previous studies showed that stereotype perception and discrimination make minority groups more susceptible to impostorism [69, 123, 124]. Additionally, we do not know the proportion of underrepresented minorities in the total population or among respondents, which might contribute to the differences observed.

Despite these limitations, this is the largest study to date that has addressed a significant knowledge gap regarding the prevalence of IP among dental students.

## 5 Conclusions

This study was the first to examine impostor phenomenon among Croatian, Bosnian and Herzegovinian dental students and found that the analyzed student populations are equally affected. It also increased our understanding of the prevalence of impostorism among dental students, showing that approximately half experience clinically relevant impostor feelings. However, there is a gender imbalance, with females more burdened by this complex behavior. Impostorism varies across the curriculum, with the transitional years 1st, 4th and 6th showing the highest average CIPS scores. Risk factors are both internal and external, and social inclusion in the broader community has a significant protective role. The IP poses a significant risk to academic performance, mental health, and retention in dental education. Although not a formal diagnosis, this aspect of the mental health continuum can disrupt training and contribute to broader psychopathology, such as depression. Dental schools should implement targeted interventions, including structured mentoring, group discussions, feedback sessions, and integrating psychological support into curricula to build student resilience. Longitudinal and epidemiological research are needed to clarify prevalence, comorbidities, and best management strategies, as current clinical evidence remains insufficient.

## Author Contributions

**Zrinka Biloglav:** conceptualization, methodology; formal analysis, data curation, visualization, writing – original draft, writing – review and editing. **Ivana Škrlec:** conceptualization, formal analysis, visualization, writing – original draft, writing – review and editing. **Petar Medaković:** formal analysis, writing – review and editing. **Ivan Padjen:** methodology, writing – review and editing. **Tatjana Ružić:** methodology, writing – review and editing. **Anđelo Kurtin:** data curation, writing – review and editing. **Vedran Jakupović:** data curation, writing – review and editing. **Džan Ahmed Jesenković:** data curation, writing – review and editing. **Simeona Olić:** data curation, writing – review and editing. **Nicole Stojanović:** methodology, data curation, writing – review and editing. **Eva Mandić:** data curation, writing – review and editing. **Emanuela Živko:** data curation, writing – review and editing. **Danijela Marović:** data curation, writing – review and editing. **Selma Jakupović:** methodology, data curation, writing – review and editing.

## Funding

The authors have nothing to report.

## Ethics Statement

The study was conducted in accordance with the Declaration of Helsinki, and approved by the Ethics Committees of the School of Dental Medicine, University of Zagreb (05‐PA‐30‐22‐11/2023, approved on 23 November 2023), and the Faculty of Dentistry, University of Sarajevo (02‐3‐4‐19‐3‐2/2024, approved on 14 May 2024).

We completed the STROBE Statement, the checklist of items that should be included in reports of cross‐sectional studies.

## Consent

Informed consent was obtained from all subjects involved in the study.

## Conflicts of Interest

The authors declare no conflicts of interest.

## Data Availability

The data presented in this study are available at the request of the corresponding author due to legal and ethical restrictions.
